# ELF3 promotes epithelial–mesenchymal transition by protecting ZEB1 from miR-141-3p-mediated silencing in hepatocellular carcinoma

**DOI:** 10.1038/s41419-018-0399-y

**Published:** 2018-03-09

**Authors:** Longbo Zheng, Ming Xu, Junjie Xu, Ke Wu, Qian Fang, Yuelong Liang, Senjun Zhou, Dong Cen, Lin Ji, Weili Han, Xiujun Cai

**Affiliations:** 1 Key Laboratory of Endoscopic Technique Research of Zhejiang Province, Department of General Surgery, Sir Run-Run Shaw Hospital, Zhejiang University, Hangzhou, 310016 China; 2 The First People’s Hospital of Wenling, Taizhou, Zhejiang China; 3 Department of Colorectal Surgery, Shaoxing People’s Hospital, Shaoxing Hospital of Zhejiang University, Shaoxing, China; 4 Department of Lung Transplantation, the First Affiliated Hospital, School of Medicine, Zhejiang University, Hangzhou, Zhejiang China

## Abstract

Hepatocellular carcinoma (HCC) is one of the most common malignant cancers and currently the third leading cause of cancer-related deaths, worldwide. Epithelial–mesenchymal transition (EMT) plays a major role in HCC progression. In this study, we first found that the expression of E74-like ETS transcription factor 3 (ELF3), a member of the E-twenty-six family of transcription factors, was increased in HCC tissues, and that ELF3 overexpression was associated with poor prognoses for HCC patients. Gain-of-function and loss-of-function studies revealed that increased ELF3 expression promoted HCC cell proliferation, migration, and invasion, while these processes were inhibited when ELF3 was silenced. Additionally, ELF3 was found to promote EMT, which we demonstrated through decreased E-cadherin expression and increased N-cadherin and fibronectin expression. ELF3 knockdown reversed EMT via repressing ZEB1 expression through miR-141-3p upregulation. Chromatin immunoprecipitation assays revealed that ELF3 bound to the miR-141-3p promoter, suppressing miR-141-3p expression. Taken together, our data show that ELF3 repressed E-cadherin and promoted EMT in HCC cells by suppressing miR-141-3p, thereby activating ZEB1. Thus, ELF3 may be a potential prognostic biomarker and/or therapeutic target for HCC.

## Introduction

Hepatocellular carcinoma (HCC) is the sixth most common human malignancy, fifth in men and eighth in women. However, HCC has one of the highest mortality rates of all cancers, accounting for approximately 1% of all cancer-related deaths worldwide^1^. Although great advancements have been made in HCC treatments, the prognosis of HCC patients remains unfavorable. These poor outcomes are primarily due to metastases and recurrences^2^.

Several intriguing studies have demonstrated that epithelial–mesenchymal transition (EMT) plays an important role in cancer metastasis^3^. EMT is a complex biological reprograming process through which epithelial cells lose their adherent features and acquire mesenchymal characteristics, such as motility, invasiveness, and resistance to apoptosis^4^. This process involves a variety of signaling pathways^5^ and is characterized by upregulated expression of mesenchymal proteins^6^. Decreased E-cadherin expression plays a key role in cancer metastasis and is one of the primary hallmarks of EMT^7^. Furthermore, it is well recognized that loss of E-cadherin and induction of EMT markers, such as fibronectin 1 and N-cadherin, in HCC tissues promotes cancer development and progression^8,9^. Additionally, transcription factors such as Snail, Slug, Twist1, Twist2, ZEB1, and ZEB2 are closely associated with EMT, as they are responsible for repressing E-cadherin expression^10,11^.

E74-like ETS tanscription factor 3 (ELF3) is a member of the epithelial-specific subfamily of ETS transcription factors, which plays a role in a variety of pathophysiologic processes including cancer and immune system disorders^12–14^. Recent studies have shown that ELF3 is involved in cancer cell proliferation, differentiation, and migration in many human tumors. ELF3 is overexpressed in colorectal cancer and promotes colorectal cancer cell proliferation and invasion by enhancing β-catenin signaling^15^. In prostate cancer, ELF3 drives tumor progression through a positive feedback loop^16^. However, the role of ELF3 in HCC is unknown.

MicroRNAs (miRNAs) are small, noncoding RNAs of 20–24 nucleotides that post-transcriptionally modulate gene expression by directly interacting with the 3′-untranslated region or 5′-untranslated region of target mRNAs^17^. Recent studies have suggested that miRNAs play important roles in tumor development, invasion, and metastasis^18,19^.

In this study, we found that ELF3 overexpression was significantly associated with poor outcomes in HCC patients, and that ELF3 could enhance HCC progression and EMT in vitro and in vivo. Mechanistic studies demonstrated that ELF3 could enhance ZEB1 expression by repressing miR-141-3p, which inhibited EMT in HCC cells.

## Materials and methods

### HCC samples and patients

The study was approved by the research ethics committee of Sir Run-Run Shaw Hospital, School of Medicine, Zhejiang University, China. Informed consent was obtained from all the patients. One hundred and six paraffin-embedded HCC samples were obtained from consecutive patients undergoing initial hepatectomy from January 2006 to December 2010 in Sir Run-Run Shaw Hospital (SRRSH), School of Medicine, Zhejiang University, China. Diagnosis of HCC was confirmed by two independent histopathologists. Tumor differentiation was graded by the Edmondson-Steiner grading system. Tumor stages were classified according to the 7th edition of the American Joint Committee on Cancer staging system. HCC clinical stages were defined based on the Barcelona Clinic Liver Cancer (BCLC) staging system^20^.

All patients were taken on 5-year follow-up. The follow-up period was defined as the interval from the date of surgery to the date of recurrence or death. The last follow-up was updated on December 10, 2010. Patients alive at the end of follow-up were censored. Study endpoint were defined as: overall survival (OS); time from liver resection to death of HCC or to the date of the last follow-up; or disease-free survival (DFS): time from the date of surgery until the detection of cancer recurrence. Patients who died from disease other than HCC or unexpected events were excluded from the study cohort.

For quantitative polymerase chain reaction (QPCR) analysis, an additional 16 pairs of fresh-frozen HCC tissue and corresponding non-tumor tissues were obtained from HCC patients undergoing initial surgery from August 2014 to December 2014 in SRRSH.

For western blot analysis, another eight pairs of fresh-frozen HCC tissues and corresponding noncancerous tissues were obtained from HCC patients undergoing initial surgery from August 2014 to December 2014 in SRRSH.

### Immunohistochemistry analysis of ELF3 and E-cadherin

Paraffin-embedded specimens were cut to 4 μm, deparaffinized with xylene, and rehydrated in a graded series of alcohols. Antigen retrieval was performed using 0.01 M citrate buffer for a 3-min boil. Hydrogen peroxide was applied to block peroxidase, and then the slides were incubated with normal goat serum. Primary antibodies were incubated overnight at 4 °C in a humidified chamber. Normal goat serum was used as a negative control. Immunohistochemistry (IHC) staining for tissues was detected by the Mo&Rb GTVision III Detection System (Gene Tech, China). DAB visualization was then performed, and the slides were counterstained with hematoxylin. Immunostaining was evaluated and scored by two pathologists without prior knowledge of the clinicopathological data using the German immunoreacted score^21^. Staining intensity was graded as “0” (negative), “1” (weak), “2” (moderate), and “3” (strong). Staining extent was graded as “0” (<5%), “1” (5–25%), “2” (25–50%), “3” (50–75%), or “4” (>75%). The intensity score was multiplied by the extent score to generate both cytoplasmic and nuclear scores (0−12), which were then combined to obtain the immunostaining score (0−24). The median of all scores was used as cutoff values for ELF3 and E-cadherin. Values ≤12 (ELF3) and ≤4 (E-cadherin) were low expression.

### Cell lines and cell culture

In this study, four human HCC cell lines (Hep3B, HepG2, MHCC-LM3, and Huh7) were purchased from the American Type Culture Collection (ATCC, Manassas, VA, USA). Cell culture was according to the manufacturer’s protocol and all the cell lines were grown at 37 °C with 5% CO_2_

### Quantitative real-time and sqRT-PCR

Total RNAs from primary tumor and adjacent non-tumor tissue (ANT) samples were extracted using TRIzol reagent (Ambion, USA) according to the manufacturer’s instructions. Complementary DNA (cDNA) was synthesized from 1 μg of RNA from each sample using iScript^TM^ cDNA Synthesis Kit (Bio-Rad, USA) or All-in-One^TM^ miRNA qRT-PCR (quantitative real-time PCR) Detection Kit (GeneCopoeia, USA). qRT-PCR was performed using a 7500 Real-time PCR System (Applied Biosystems Inc., USA). Each experiment was conducted with at least three independent replicates. Relative quantification of mRNA expression was calculated using the ∆∆Ct method. Glyceraldehyde 3-phosphate dehydrogenase was used as an internal control. Primer sequences are listed in the Supplementary Table 1.

### Western blot analysis

Total proteins were extracted with RIPA lysis buffer and separated by sodium dodecyl sulfate-polyacrylamide gel electrophoresis and then transferred to the PVDF membrane (Millipore, USA) and incubated overnight at 4 °C with appropriate antibody. The antigen–antibody complex on the membrane was detected with enhanced chemiluminescence regents (Thermo Scientific, Waltham, MA, USA). The antibodies are listed in the Supplementary Table 2.

### Transwell migration and invasion assay

For the migration assay, about 1 × 10^5^ cells suspended in serum-free media were placed in the upper chamber. For the invasion assay, the membrane was coated with Matrigel (BD Biosciences, USA) to form a matrix barrier, and then 1 × 10^5^ cells were placed in the upper chamber. In each lower chamber, 600 ml of Dulbecco's modified Eagle's medium with 10% fetal bovine serum was added. After 48 h of incubation at 37 °C, the cells in the lower compartment were stained with 0.1% crystal violet and counted in five randomly chosen fields (100×) under a microscope. The experiment was performed with three replicates.

### Cell counting kit-8 assay and colony formation assay

A total of 3 × 10^3^ cells were dispensed into per well of the 96-well plates in a final volume of 100 μl. The plates were incubated at 37 °C for 24 h in a humidified 5% CO_2_ atmosphere. Ten microliters of the CCK-8 solution was added to each well of the plate, and then incubated for 2 h in an incubator. The absorbance was measured at 450 nm using a 96-well plate reader. Each experiment was conducted with three replicates.

For colony formation assays, cells were seeded into 6-well plate at a density of 1 × 10^3^ cells and cultured for 2 weeks at 37 °C. The number of colonies per well was counted after staining with crystal violet. The colonies with >50 cells under the microscope were counted. All studies were conducted with three replicates.

### Oligonucleotide transfection

Small interference RNA (siRNA) targeting ELF3, ZEB1 (siELF3, siZEB1), negative control siRNA (sicontrol), miRNA inhibitor (miR-141-3p, miR-494-3p), and negative control miRNA were obtained from Ribobio (Guangzhou, China). siRNAs, miRNA inhibitors, and negative controls were transient transfected into HCC cells at a working concentration of 50 nM using Lipofectamine 2000 reagent (Invitrogen, USA) according to the manufacturer’s protocol. Gene silencing effect was confirmed by qPCR and western blot at 48–72 h post transfection. Sequences of miRNA inhibitors and siRNAs are listed in Supplementary Table 3.

### Establishment of ELF3 overexpression and knockdown cells

ELF3 ectopic expression and knockdown lentivirus as well as their negative control lentivirus were purchased from GeneChem (Shanghai, China). Full-length human ELF3 ectopic expression lentivirus was transfected into Huh7 cells. Lentivirus containing short hairpin RNAs (shRNAs) targeting ELF3 was transfected into LM3 cells according to the manufacturer’s instructions. The sequences of RNAi and cDNA clones are listed in Supplementary Table 3.

### Chromatin immunoprecipitation and CHIP-qPCR assay

Chromatin immunoprecipitation (CHIP) assays were performed using CHIP Assay Kit (P2078, Beyotime, China) according to the manufacturer’s protocol. Briefly, MHCC-LM3 cells were collected and fixed for 10 min at 37 °C with 1% formaldehyde, followed in sequence with SDS lysis and DNA shearing, protein and DNA immunoprecipitation, cross-linked DNA reversal and DNA purification, and finally the immunoprecipitated DNA fragments were detected by real-time PCR assays and qPCR. The normal rabbit IgG was used as the negative control. The primers were listed in Supplementary Table 1. The CHIP and CHIP-qRT-PCR were performed with three replicates.

### Luciferase assays

The miR-141 promoter and the mutant promoter were purchased from GeneChem (Shanghai, China). MHCC-LM3-shELF3 and MHCC-LM3-shcontrol cells were seeded into 6-well tissue plates 24 h before transfection, and then transfected with cDNA transfected using Lipofectamine 2000 (Invitrogen, USA) according to the manufacturer’s instruction. pRL-TK was used as an internal control. Luciferase activity was measured by Dual-Luciferase Assay (Promega) according to the manufacturer’s manual. All experiments were performed in triplicated and data were pooled from three independent experiments. The detailed sequences of wild-type and mutant promoter could be found in Supplementary Table 4.

### Xenograft growth assay

To assess the function of the ELF3 in HCC growth in vivo, 5 × 10^6^ cells from transfected MHCC-LM3-shELF3 cells and their control cells were implanted subcutaneously into 4-week-old nude mice. The subcutaneous tumor size was calculated and recorded every week using the Vernier caliper as follows: tumor volume (mm^3^) = (*L*x*W*^2^)/2, where *L*is the long axis and *W* the short axis. The subcutaneous tumor tissues were removed and calculated 5 weeks later.

### Metastasis analysis in a mouse model

In vivo metastasis experiments were done using a 4-week-old male BALB/c nu/nu mice. In vivo metastasis assays were performed as described previously. Mice were anesthetized by pentobarbital, and spleens exteriorized via a flank incision. LM3 cells that stably express firefly luciferase (Xenogen) were infected with lentiviruses carrying shELF3 and control shRNA. LM3 were injected into the lower polar side of the spleen through a 27-gauge needle. For bioluminescence, IVIS (in vivo imaging system) Spectrum Image System (Xenogen, Alameda, CA, USA) was used. Liver were fixed in formalin and embedded in paraffin blocks for slicing into thin sections. The paraffinized sections were stained with hematoxylin and eosin according to standard protocols.

### Statistical analysis

Statistical analyses were performed using SPSS 20 for Windows and GraphPad Prism 5. Data were expressed as the mean ± standard error of the mean (SEM) from at least three independent experiments. Quantitative data between groups were compared using the Student's *t* test. Correlations between different protein expressions level were determined using Spearman’s rank analysis. OS and DFS curves were obtained by the Kaplan–Meier method, and differences were compared by the log-rank test. Univariate analysis and multivariate analysis were analyzed with Cox proportional hazard regression model to verify the independent risk factors. A two-tailed *P* value of <0.05 was considered as statistical significance.

## Results

### ELF3 is upregulated in HCC

**Fig. 1:**
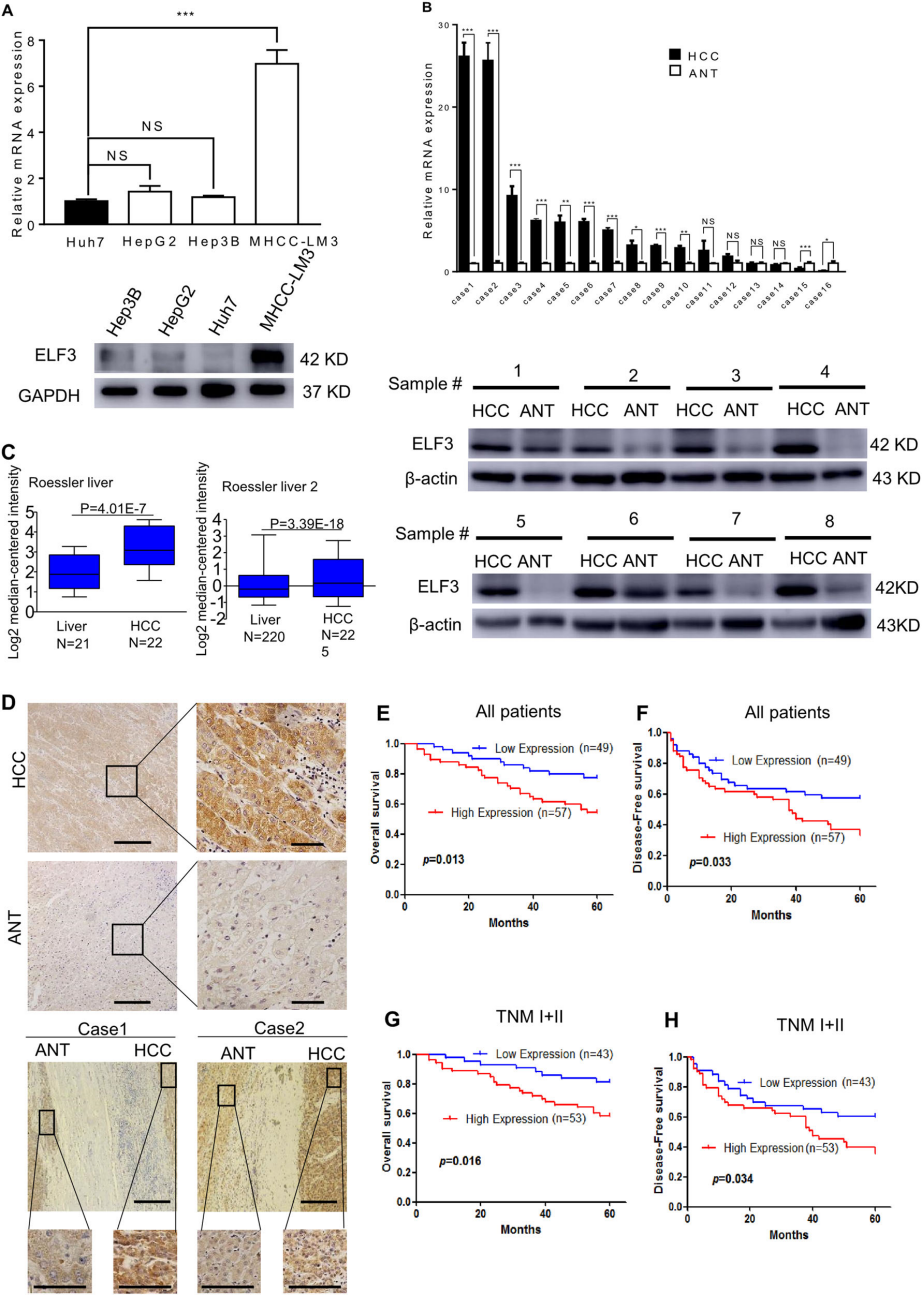
Expression of analyses of ELF3 mRNA and protein in HCC cell lines and tissues. **a** ELF3 expression in four HCC cell lines analyzed by qPCR and western blot. **b** ELF3 expression is upregulated in HCC tissues analyzed by qPCR and western blot. **c** Upregulation of ELF3 expression in HCC tissues at the mRNA level compared with normal liver tissues revealed using the two Roessler liver datasets from the Oncomine dataset. **d** Representative IHC image of ELF3 expression in HCC and ANT (magnification ×100, scale bar:200μm). The region in the black square was further enlarged (magnificantion ×400, scale bar:50μm). OS and DFS of HCC patients with low and high ELF3 expression in all HCC patients (**e**, **f**) and HCC patients with early-stage tumor (**g**, **h**). Survival curve was calculated with the log-rank test. Each error bar represents the mean ± SD of three replicate samples. *p* < 0.05 was considered statistically significant. *p < 0.05, **p < 0.01 based on the Student's *t* test

Western blot and qRT-PCR assays were performed to evaluate ELF3 expression in HCC cell lines. As shown in Fig. 1a, ELF3 was expressed highest in MHCC-LM3 cells and lowest in Huh7 cells. ELF3 expression was also analyzed by western blot and qRT-PCR in HCC tissues and corresponding ANTs. These data showed that ELF3 expression was much higher in HCC tissues than in ANTs (Fig. 1b). We then analyzed *ELF3* mRNA expression in the Oncomine database (www.oncomine.org). This analysis also showed higher ELF3 expression in HCC tissues than paired noncancerous tissues; similar results were found using Roessler Liver Datasets 1 and 2 (Fig. 1c). Moreover, IHC showed higher ELF3 expression in HCC tissues than ANTs (Fig. 1d). These results all confirmed that ELF3 was upregulated in HCC.

### Association between ELF3 expression and clinicopathological features

To understand how ELF3 overexpression affects HCC development, we evaluated associations between ELF3 expression and clinicopathological features and survival data of HCC patients. As shown in Table 1, ELF3 overexpression was significantly associated with tumor size (*p* < 0.05) (Table 1). To estimate associations between ELF3 expression and the postsurgical prognoses of HCC patients, 5-year OS and DFS rates were analyzed by Kaplan–Meier analysis and log-rank test. HCC patients with higher ELF3 expression had worse OS (*p* = 0.013) (Fig. 1e) and DFS (P = 0.033) (Fig. 1f). Similarly, early-stage (stage I + II) HCC patients with higher ELF3 expression had worse OS (*p* = 0.016) (Fig. 1g) and DFS (*p* = 0.034) (Fig. 1h).

**Table 1:** Association between ELF3 expression and clinicopathology feature in patients with HCC

Variable	ELF3 expression		*p*
	Low (%)	High (%)	
Age			
<60 years	20 (40.8)	28 (49.1)	0.392
≥60 years	29 (59.2)	29 (50.9)	
Gender			
Male	40 (81.6)	48 (84.2)	0.725
Female	9 (18.4)	9 (15.8)	
HBsAg			
Negative	5 (10.2)	12 (21.1)	0.129
Positive	44 (89.8)	45 (78.9)	
AFP (ng/mL)			
<400	29 (59.2)	38 (66.7)	0.426
≥400	20 (40.8)	19 (33.3)	
Liver cirrhosis			
No	25 (51.0)	25 (43.9)	0.462
Yes	24 (49.0)	32 (56.1)	
Tumor size			
<5 cm	35 (71.4)	30 (52.6)	0.048
≥5 cm	14 (28.6)	27 (47.4)	
Tumor number			
Single	43 (87.8)	51 (89.5)	0.781
Multiple	6 (12.2)	6 (10.5)	
Tumor differentiation			
Well/moderately	21 (42.9)	33 (57.9)	0.123
Poorly	28 (57.1)	24 (42.1)	
Tumor thrombi			
No	41 (83.7)	51 (89.5)	0.379
Yes	8 (16.3)	6 (10.5)	
TNM stage*			
I + II	43 (87.8)	53 (93.0)	0.508
III + IV	6 (12.2)	4 (7.0)	
BCLC stage			
A	44 (89.8)	52 (91.2)	1.000
B	5 (10.2)	5 (8.8)	

*HCC *hepatocellular carcinoma, *HBsAg* hepatitis B surface antigen, *AFP* α-fetoprotein, *TNM* tumor node metastasis, *BCLC* Barcelona Clinic Liver Cancer

To further evaluate whether ELF3 expression was an independent risk factor for HCC patients, univariate and multivariate analyses were performed. The univariate results indicated that ELF3 overexpression was significantly correlated with OS and DFS in HCC patients (Table 2). Multivariate analysis revealed that ELF3 was an independent risk factor for HCC (OS: hazard ratio (HR) = 2.66, 95% confidence interval (95% CI): 1.28–5.55, *p* = 0.009; DFS: HR = 1.98, 95% CI: 1.13–3.47, *p* = 0.017) (Table 2). These findings demonstrated that ELF3 might be a new prognostic biomarker for HCC.

**Table 2:** Univariate and multivariate analyses of prognostic factors for overall survival and disease-free survival of HCC patients in the study cohort

Variable	OS				DFS			
	Univariate		Multivariate		Univariate		Multivariate	
	HR (95% CI)	*p*	HR (95% CI)	*p*	HR (95% CI)	*p*	HR (95% CI)	*p*
Age								
≥60 years	0.87 (0.46–1.66)	0.672			0.69 (0.41–1.15)	0.154		
Gender								
Female	0.83 (0.36–1.89)	0.655			1.28 (0.61–2.70)	0.515		
HBsAg								
Yes	0.83 (0.36–1.89)	0.655			1.13 (0.56–2.30)	0.732		
AFP (ng/mL)								
≥ 400	1.56 (0.81–2.99)	0.182			1.16 (0.68–1.98)	0.581		
Liver cirrhosis								
Yes	1.25 (0.65–2.40)	0.496			1.44 (0.86–2.43)	0.166		
Tumor size								
≥5 cm	2.33 (1.22–4.46)	0.010	1.65 (0.81–3.37)	0.169	2.22 (1.33–3.71)	0.002	1.95 (1.11–3.43)	0.021
Tumor number								
Multiple	1.04 (0.37–2.93)	0.943			1.66 (0.79–3.51)	0.182		
Tumor differentiation								
Poorly	1.30 (0.68–2.47)	0.433			0.89 (0.54–1.49)	0.668		
Tumor thrombi								
Yes	2.31 (1.05–5.05)	0.037	1.69 (0.70–4.12)	0.246	3.68 (1.94–7.01)	<0.001	3.50 (1.75–6.99)	<0.001
TNM stage								
III + IV	3.48 (1.52–7.99)	0.003	2.73 (0.96–7.77)	0.059	3.12 (1.47–6.62)	0.003	1.91 (0.77–4.72)	0.161
Group								
High	2.38 (1.17–4.81)	0.016	2.66 (1.28–5.55)	0.009	1.76 (1.03–3.01)	0.037	1.98 (1.13–3.47)	0.017

*HCC* hepatocellular carcinoma, *OS* overall survival, *DFS* disease-free survial, *HBsAg* hepatitis B surface antigen, *AFP* α-fetoprotein, *TNM* tumor node metastasis, *HR* hazard risk ratio, *CI* confidence interval

### ELF3 overexpression promotes the proliferation, migration, and invasion of HCC cells

**Fig. 2:**
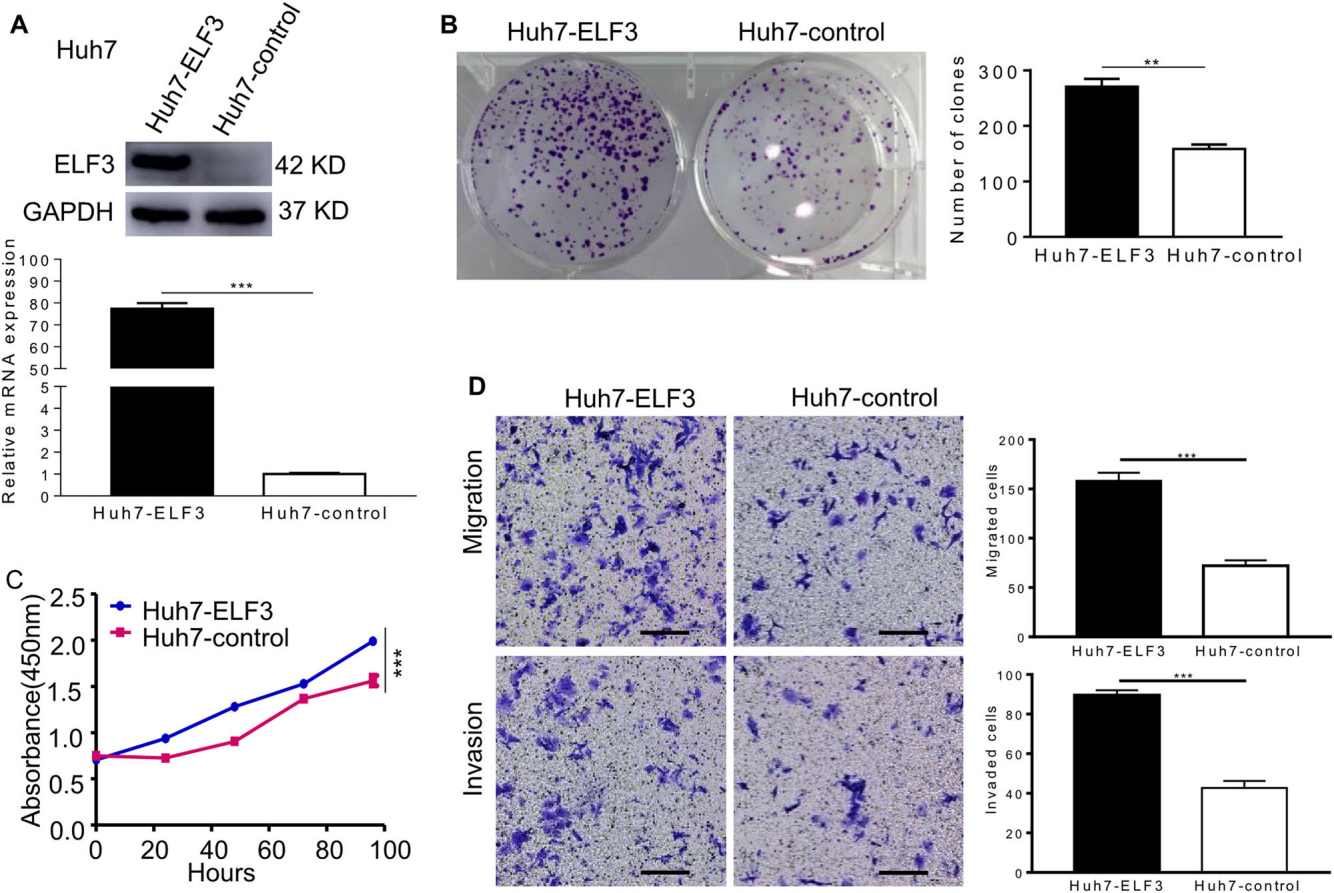
ELF3 overexpression promoted the proliferation, migration, and invasion of HCC cell in vitro. **a** Western blot and qPCR evaluated the protein expression and mRNA of ELF3 in Huh7-ELF3 cells and Huh7-control cells. **b** The colony formation and **c** CCK-8 assay revealed that ELF3 overexpression promoted cell growth in vitro. **d** Transwell migration and invasion assays indicated that ELF3 ectopic expression promoted cell migration and invasion (magnification ×100, scale bar: 200 μm). Each error bar represents the mean ± SD of three replicate samples. *p* < 0.05 was considered statistically significant. *p < 0.05, **p < 0.01, and ***p < 0.001 based on the Student's *t* test

To further investigate the functional role of ELF3 in HCC cells, we established stable ELF3-overexpressing Huh7 cells (Huh7-ELF3). Huh7 cells infected with empty lentiviral vectors (Huh7-con) were used as negative control cells. Western blot and qRT-PCR analyses showed a significant increase in ELF3 expression in Huh7-ELF3 compared with Huh7-con cells (Fig. 2a). In colony formation assays, Huh7-ELF3 cells formed more colonies (Fig. 2b). Consistently, Huh7-ELF3 cells showed a higher proliferation rate than Huh7-con cells (Fig. 2c). Finally, Huh7-ELF3 cells showed remarkably higher migration and invasion rates than Huh7-con cells (Fig. 2d).

### Silencing *ELF3* represses the proliferation, migration, and invasion of HCC cells

**Fig. 3:**
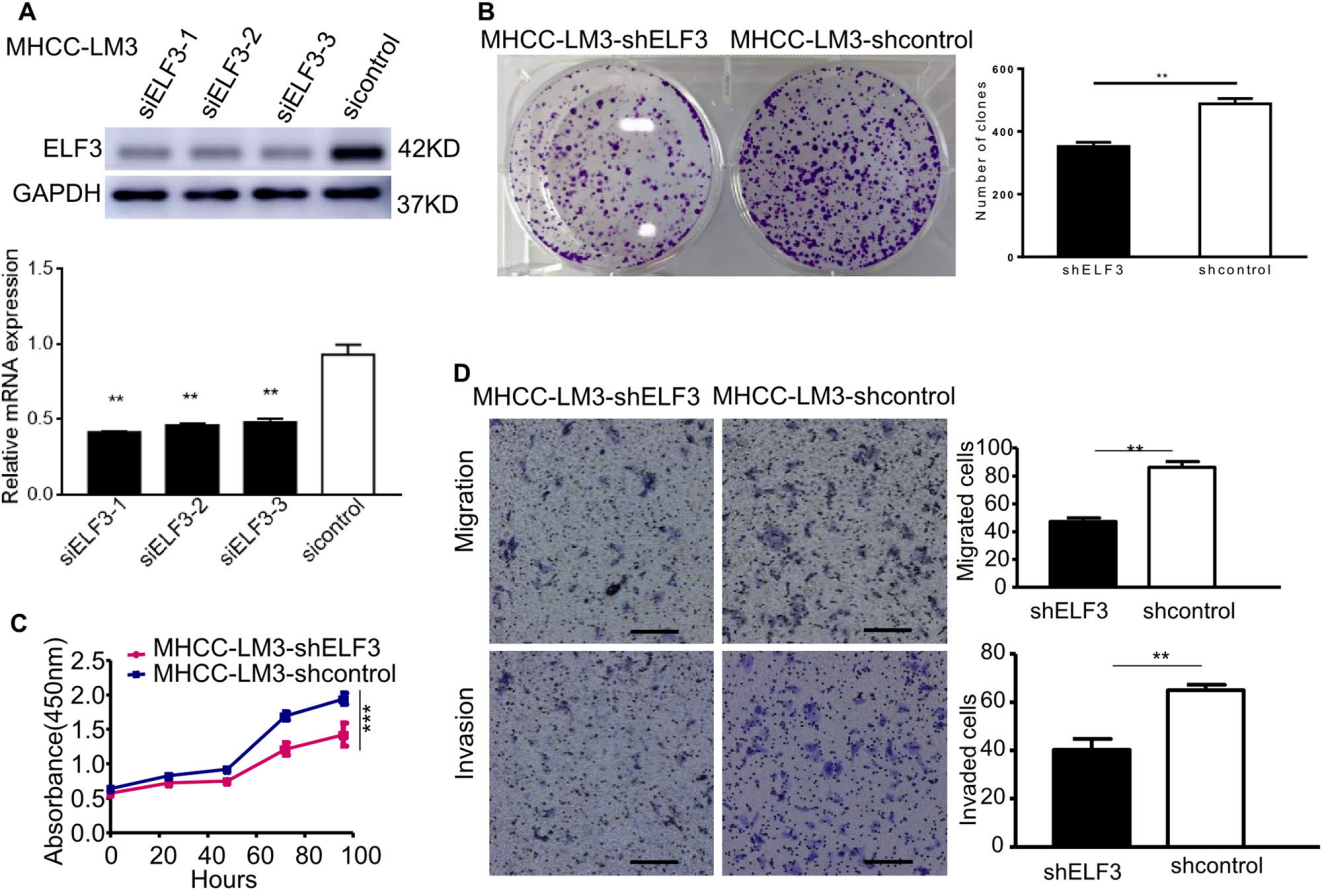
Down-regulation of ELF3 inhibited the proliferation, migration, and invasion of HCC cell in vitro. **a** Western blot and qPCR evaluated the protein expression and mRNA of ELF3 in MHCC-LM3-siELF3-1, MHCC-LM3-siELF3-2, MHCC-LM3-siELF3-3 cells and MHCC-LM3-sicontrol cells. **b** The colony formation and **c** CCK-8 assay revealed that down-regulation of ELF3 inhibited MHCC-LM3-shELF3 cells' growth in vitro. **d** Transwell migration and invasion assays indicated that ELF3 knockdown inhibited cell migration and invasion (magnification ×100, scale bar: 200 μm). Each error bar represents the mean ± SD of three replicate samples. *p* < 0.05 was considered statistically significant. *p < 0.05, **p < 0.01, and ***p < 0.001 based on the Student's *t* test

To further evaluate the function of ELF3 in HCC cells, three siRNAs (siELF3-1, siELF3-2, and siELF3-3) were designed to silence ELF3 expression in MHCC-LM3 cells. ELF3 expression was verified by qRT-PCR and western blot, and siELF3-1 was found to be the most effective siRNA and was chosen for subsequent studies (Fig. 3a). Next, shELF3, a lentiviral vector carrying the siELF3-1 sequence, was designed to knockdown ELF3 expression in MHCC-LM3 cells (MHCC-LM3-shELF3). Compared with MHCC-LM3-shcon and MHCC-LM3-shELF3 cells had significantly reduced proliferation and colony formation rates (Fig. 3b, c).

Next, we investigated the role of ELF3 in tumor growth in vivo. We performed xenograft growth assays in nude mice following subcutaneous injection of MHCC-LM3-shELF3 and MHCC-LM3-shcon cells. Tumors from the MHCC-LM3-shELF3 group were significantly smaller than those from the control group on day 35 (*p* < 0.05; Supplementary Figure 1A). Transwell assays also showed that ELF3 knockdown significantly reduced the migratory and invasive ability of Huh7 cells (Fig. 3d).

### ELF3 promotes EMT by regulating ZEB1 activation in HCC cells

**Fig. 4:**
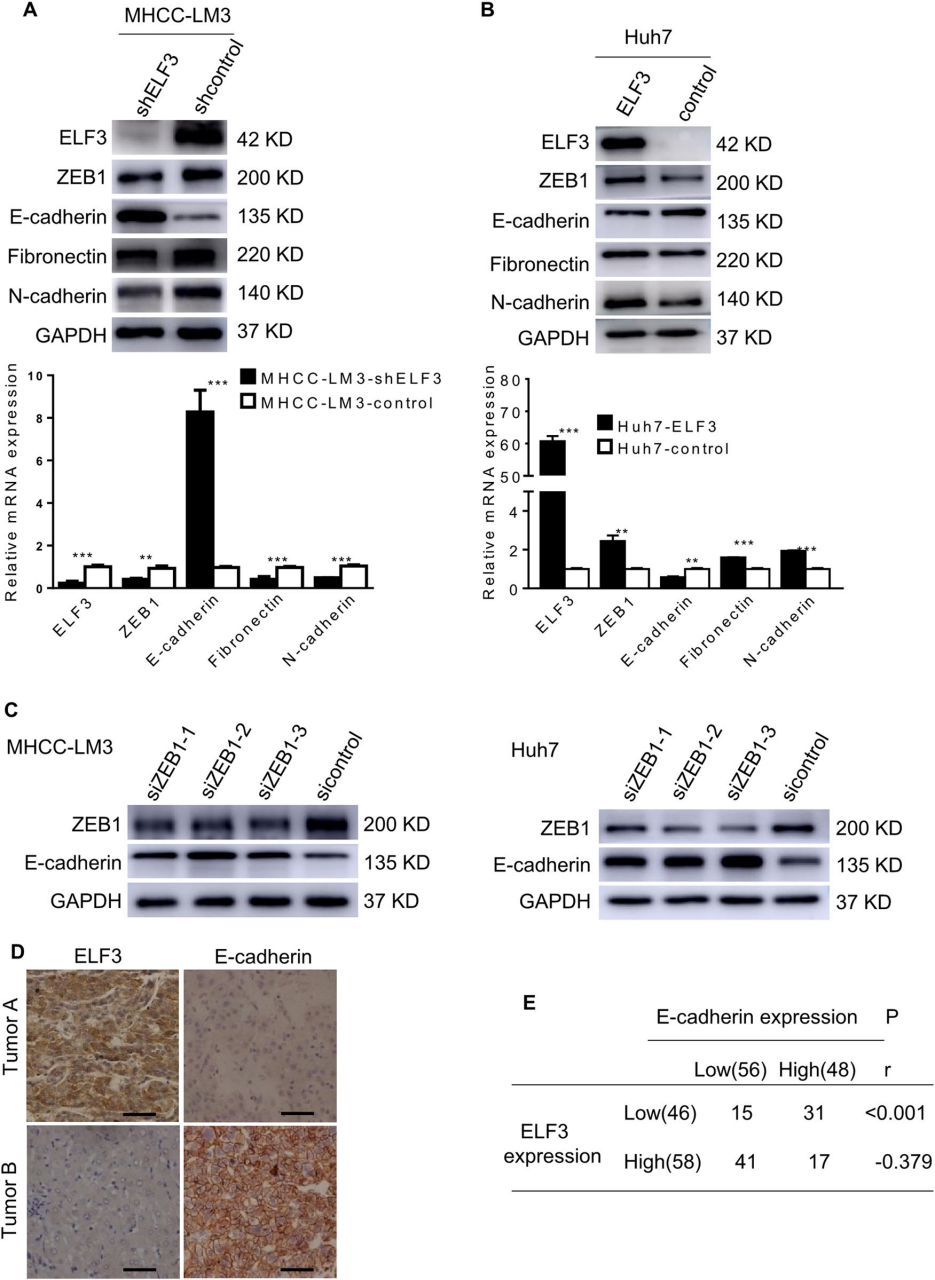
ELF3 promotes EMT by regulating ZEB1 activation in HCC cells. **a** mRNA and protein expression of ELF3, E-cadherin, ZEB1, fibronectin, N-cadherin in MHCC-LM3-shELF3 cells and in Huh7-ELF3 cells (**b**) and their control cells were analyzed by qPCR and western blot. **c** Protein expression of E-cadherin and ZEB1 in MHCC-LM3 and Huh7 cells with ZEB1 knockdown were analyzed by western blot. **d** Representative IHC images of ELF3 and E-cadherin expression in HCC tissues (magnification ×400, scale bar: 50 μm). **e** The correlation analysis between ELF3 and E-cadherin in HCC samples. Each error bar represents the mean ± SD of three replicate samples. *p* < 0.05 was considered statistically significant. *p < 0.05, **p < 0.01, and ***p < 0.001 based on the Student's *t* test

To further explore the possible mechanisms of ELF3-induced EMT in HCC cells, we focused on the expression of EMT-associated markers. Western blot and qRT-PCR analyses showed that silencing ELF3 in MHCC-LM3 cells increased E-cadherin expression and decreased the expression of N-cadherin, fibronectin, and ZEB1 (an EMT transcriptional factor) (Fig. 4a), whereas upregulating ELF3 in Huh7 cells induced the opposite results (Fig. 4b). However, western blots for ZEB2, SLUG, and SNAIL showed no significant change after manipulating ELF3 expression (Supplementary Figure 1B). To determine whether ZEB1 was involved in EMT in HCC cells, we silenced ZEB1 using three siRNAs in MHCC-LM3 and Huh7 cells. We found that ZEB1 knockdown in MHCC-LM3 and Huh7 cells increased E-cadherin expression (Fig. 4c). An analysis of the Gene Expression Profiling Interactive Analysis (GEPIA) database^22^ revealed a positive correlation between ELF3 and ZEB1 (Supplementary Figure 1C). These results suggested that ELF3 regulated ZEB1 at the transcriptional level in a direct or indirect manner. However, CHIP assays demonstrated that ELF3 increased ZEB1 indirectly (Supplementary Figure 1D). Additionally, IHC was performed to analyze a possible correlation between ELF3 and E-cadherin expression in HCC tissues. As shown in Fig. 4d, e, 31/46 HCC samples (67.4%) with low ELF3 expression were found to have high E-cadherin expression, whereas only 29.3% of specimens with high ELF3 expression had high E-cadherin expression. Spearman's correlation analysis showed that ELF3 expression was negatively correlated with E-cadherin expression in HCC samples (Fig. 4e, *p* < 0.001, *r* = −0.379). These results suggested that ELF3-induced EMT by indirectly activating ZEB1.

### ELF3 knockdown decreased ZEB1 levels by upregulating miR-141-3p

**Fig. 5:**
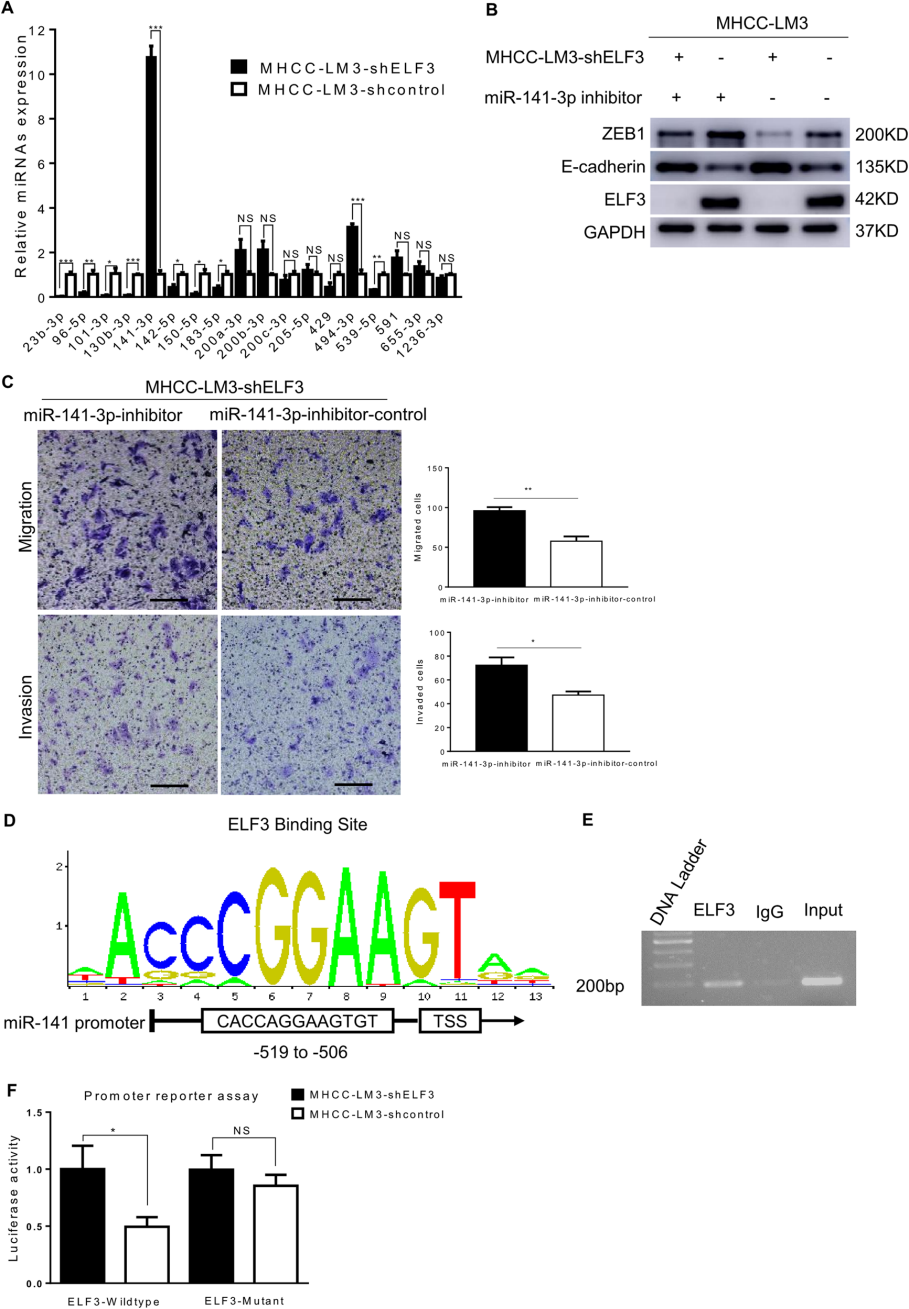
Knockdown of ELF3 decreased ZEB1 by upregulating miR-141-3p. **a** miRNAs of expression in MHCC-LM3-shELF3 cells and MHCC-LM3-shcontrol cells were analyzed by sqRT-PCR. **b** Protein expression of ZEB1 and E-cadherin in MHCC-LM3-shELF3 cells and MHCC-LM3-shcontrol cells with miR-141-3p inhibitor or control were analyzed by western blot. **c** Migration and invasion assay indicated that 141-3p inhibitor increased MHCC-LM3-shELF3 cells' migration and invasion (magnification ×100, scale bar: 200 μm). **d** Predicted ELF3 binding site in miR-141-3p promoter from JASPER. **e** CHIP assay showed that ELF3 could bind to the promoter of miR-141-3p. **f** promoter reporter assay and mutation rescue assay suggested that ELF3 could inhibit miR-141 promoter activity by specifically interacting with the predicted genomic region. Each error bar represents the mean ± SD of three replicate samples. *p* < 0.05 was considered statistically significant. *p < 0.05, **p < 0.01, and ***p < 0.001 based on the Student's *t* test

It has been reported that many miRNAs target *ZEB1* mRNA to repress ZEB1 expression^23–25^. Thus, we investigated whether miRNAs were involved in ELF3-dependent EMT. We first searched miRTarBase and found 18 miRNAs that could directly target ZEB1. To systemically screen amongst this group for miRNAs regulated by ELF3, miRNA-specific qRT-PCR was performed. ELF3 knockdown significantly enhanced the expression of miR-141-3p and miR-494-3p in MHCC-LM3 cells, and ELF3 attenuated miR-141-3p and miR-494-3p activity in Huh7 cells (Fig. 5a and Supplementary Figure 1E). Western blots assays were then performed on these two selected candidates. The results showed that the miR-141-3p inhibitor upregulated ZEB1 expression and decreased E-cadherin expression more significantly in MHCC-LM3-shELF3 and MHCC-LM3-con cells compared with the miR-141-3p control inhibitor (Fig. 5b). However, the miRNA-494-3p inhibitor had no significant effects on ZEB1 levels (Supplementary 1F). Transwell assays showed that the miR-141-3p inhibitor increased the migration and invasion of both MHCC-LM3-shELF3 cells (Fig. 5c) and MHCC-LM3-shcon cells (Supplementary Figure 2A). To rule out off-target effects, we measured pri-miR-141 and pre-miR-141 levels in MHCC-LM3-shELF3 cells, Huh7-ELF3 cells, and their respective controls. These results were consistent with miR-141-3p (Supplementary Figure 2B-C).

As the miR-200 family are transcribed as two independent primary transcripts, and miR-141 is located downstream of miR-200c on the miR-200c/141 cluster^23^, but ELF3 suppresses only miR-141-3p, we sought to determine whether ELF3 regulates pri-miR-200c and pre-miR-200c. We found that pri-miR-200c and pre-miR-200c were not significantly changed after altering ELF3 expression (Supplementary Figure 2D, E). Next, we used the JASPAR Database to predict putative ELF3 binding sites in the miR-141 promoter region. This analysis showed a highly positive ELF3 binding site upstream (−519 to −506) of the transcriptional start site (Fig. 5d). CHIP assays in MHCC-LM3 cells showed that ELF3 could physically bind the miR-141 promoter (Fig. 5e). Moreover, promoter reporter and mutant rescue assays further suggested that ELF3 inhibited miRNA-141-3p promoter activity (Fig. 5f). Additionally, we performed CHIP-qRT-PCR analyses in MHCC-LM3 cells including the genomic region upstream (−819 to −519) and downstream (−506 to −206) of the ELF3 binding site, using IgG as a control (Supplementary 2F). These data demonstrated that ELF3 could directly target miR-141-3p, promoting EMT in HCC.

### ELF3 enhances HCC metastasis in vivo

**Fig. 6:**
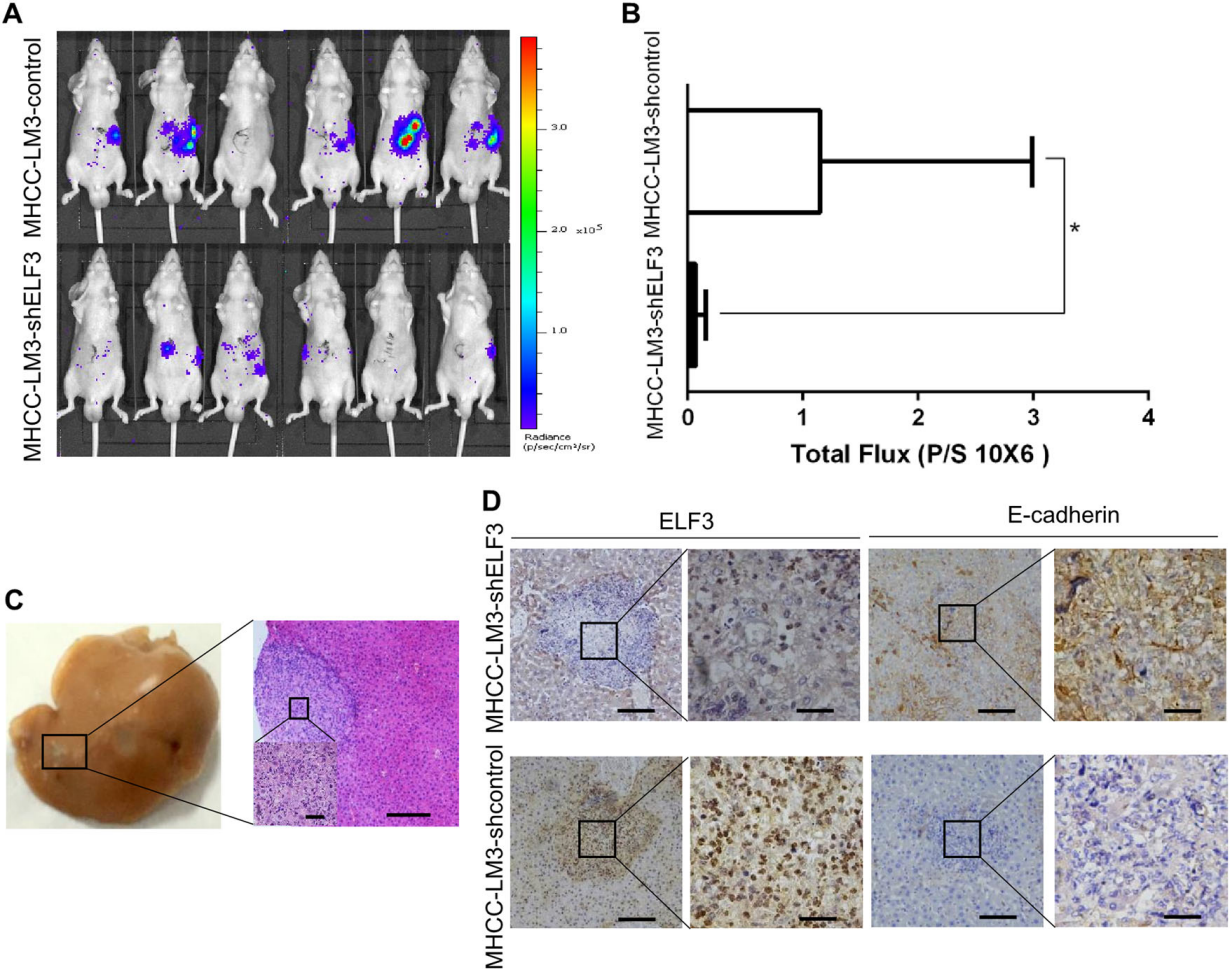
Knockdown of ELF3 inhibited MHCC-LM3 cell metastasis in vivo. **a** IVIS images of BALB/c nu/nu mice injected with MHCC-LM3-shELF3 cells and MHCC-LM3-shcontrol cells. **b** The total photon flux were measured and analyzed using the Living Image software, and the results showed less total metastatic foci in the MHCC-LM3-shELF3 group. **c** Representative images of hematoxylin and eosin staining of xenograft tumors (magnification ×100, scale bar:200μm). The region in the black square was further enlarged (magnificantion ×400, scale bar:50μm). **d** Representative IHC images of ELF3 and E-cadherin in xenograft tumors (magnification ×100, scale bar:200μm). The region in the black square was further enlarged (magnification ×400, scale bar:50μm). *p* < 0.05 was considered statistically significant. *p < 0.05, **p < 0.01, and ***p < 0.001 based on the Student *t* test

To further investigate the role of ELF3 in HCC metastasis in vivo, MHCC-LM3 cells stably expressing luciferase (MHCC-LM3-luc) were infected with ELF3 or control shRNA to establish stable HCC cell lines. The effect of ELF3 on tumor metastasis was assessed in immunocompromised male BALB/c mice (*n* = 6), using the splenic injection model^26^. Tumor cell dissemination was monitored weekly by IVIS imaging. After 4 weeks, the results showed that liver metastases were significantly reduced in mice injected with MHCC-LM3-luc-shELF3 cells compared with mice injected with MHCC-LM3-luc-con cells (Fig. 6a). Significantly, we also demonstrated less total metastatic foci in the MHCC-LM3-shELF3 group (Fig. 6b). Metastatic tumor nodules in the liver were confirmed by hematoxylin and eosin staining (Fig. 6c). ELF3 and E-cadherin expression in xenograft tumors were verified by IHC. As shown in Fig. 6d, higher ELF3 expression was associated with lower E-cadherin expression, and vice versa in MHCC-LM3-shELF3 cells.

## Discussion

HCC is one of the most common and fatal malignancies in the world^27^. Because of its tendency to invade blood vessels, resulting in intrahepatic and extrahepatic metastases, the outcomes of HCC patients after surgical resection still remain unsatisfactory^28,29^. Previous studies have revealed that EMT, an essential phenotypic conversion during embryonic development, plays an important role in cancer progression and metastasis^7,30,31^. The acquisition of EMT features not only increases the invasive and metastatic properties of cancer cells but also gives rise to postsurgical recurrence and metastasis. In this study, we first demonstrated that ELF3 showed abnormally high expression in HCC, and that its biological activity was correlated with EMT phenotypes in HCC cell lines in vitro.

ELF3 was discovered more than 20 years ago, and our understanding of its functions in tumor cells has grown exponentially over the past decade. ELF3 is expressed in epithelial cells and plays a critical role in the earliest stages of embryogenesis^32^. Loss of ELF3 in epithelial cells results in epithelial cell differentiation and cancer, as well as many other diseases^33^. These characteristics are similar to the biological functions of EMT. Previous studies have revealed that ELF3 plays important roles in invasion, migration, and tumor progression^15,16,34^; however, recent evidence indicated that ELF3 overexpression suppressed EMT in ovarian cancer^35^. In this study, we found that ELF3 was overexpressed in HCC tissues compared with ANTs. In our analysis, high-level ELF3 expression was an independent prognostic marker for HCC patients. More interestingly, survival analyses of early-stage HCC patients revealed that patients with higher ELF3 expression had shorter OS and DFS. Gain-of-function and loss-of-function experiments revealed that ELF3 overexpression strongly promoted the migration, invasion, and proliferation of HCC cells, while ELF3 knockdown yielded the opposite results. Taken together, these data suggested that ELF3 may serve as a marker associated with poor prognosis and a high risk of tumor metastasis in HCC.

We further examined the mechanism through which ELF3 promoted EMT in HCC cells. ELF3 expression was associated with known EMT markers, including E-cadherin, N-cadherin, and fibronectin. We then identified the specific EMT regulators involved in ELF3-induced EMT. Transcription factors that regulate E-cadherin, such as Snail, Slug, Twist, ZEB1, and ZEB2, are known to control EMT^36,37^. Among these transcription factors, we found that ELF3-induced EMT through ZEB1 activation. ZEB1, a member of the ZEB family of transcription factors, has been reported to be overexpressed in several human cancers, including HCC^38–40^. Previous studies have shown that ZEB1 directly promotes EMT in HCC. Consistent with these studies, we found that ZEB1 knockdown increased E-cadherin expression. However, we found that ELF3 did not directly bind to the ZEB1 promoter. Taken together, these results provided the first evidence that ELF3 promoted EMT in HCC by indirectly activating ZEB1.

To understand the relationship between ELF3 and ZEB1, we evaluated correlations between miRNAs and ZEB1, as previous studies had shown that ZEB1 is regulated by several pathways, including miRNAs^24,41,42^. Several miRNAs, including the miR-200 family^43–46^, miR-205^24^, miR-150^23^, and miR-130b^47^, have been shown to directly repress ZEB1. Our results indicated that ELF3 was an miR-141-3p suppressor that directly bound to the miR-141-3p promoter. It has been well documented that miR-141 plays important roles in human cancers. MiR-141-3p is a member of the miR-200 family, which together is considered an important negative regulator of EMT^48^. A previous study suggested that miR-141 inhibited the migration and invasion of Madin–Darby canine kidney epithelial cells^24^. The miR-200 family has five members, which are transcribed as two primary transcripts (miR-200b-200c-429 and miR-200a-141)^49^. Although miR-141 and miR-200c are in the same cluster, these two miRNAs are not always co-expressed^50,51^. In this study, we found that miR-141, but not miR-200c, was upregulated in MHCC-LM3-shELF3 cells, which further supported differential regulation of miRNAs located in the same cluster. Together, these results suggest that miRNA-141-3p plays a key role in ELF3-induced EMT and is an ELF3 target in HCC cells.

In conclusion, our findings first revealed that ELF3 was a prognostic indictor in HCC patients. Moreover, in vivo and in vitro assays showed that ELF3 promoted EMT by activating ZEB1; silencing ELF3 downregulated ZEB1 and upregulated E-cadherin by relieving the inhibition of miR-141-3p expression. Our discoveries highlight ELF3 as a promising biomarker and therapeutic target for HCC.

## Electronic supplementary material

Supplementary

Supplementary Figure 1

Supplementary Figure 2
