# Supplementary material for: ELF3 promotes epithelial–mesenchymal transition by protecting ZEB1 from miR-141-3p-mediated silencing in hepatocellular carcinoma
Source: Cell Death Dis. 2018 Mar 9;9(3):387. doi: 10.1038/s41419-018-0399-y (PMC5845010; doi:10.1038/s41419-018-0399-y)
Supplement: Supplementary file 1 — Supplementary [file 41419_2018_399_MOESM1_ESM.docx]

| Supplementary Table 1. The sequence of PCR primers and CHIP-RT-PCR used in this study | | |
| --- | --- | --- |
| Target gene | Application | Sequence |
| ELF3 | qRT-PCR | F: GGCCCAGACCAAGCCTTAAT |
|  |  | R: CACTGAAAGCCAGGGCAAAC |
| E-cadherin | qRT-PCR | F: AAGTGCTGCAGCCAAAGACAGA |
|  |  | R: AAATTGCCAGGCTCAATGACAAG |
| N-cadherin | qRT-PCR | F: AGCCAACCTTAACTGAGGAGT |
|  |  | R: GGCAAGTTGATTGGAGGGATG |
| Fibronectin | qRT-PCR | F: CGGTGGCTGTCAGTCAAAG |
|  |  | R: AAACCTCGGCTTCCTCCATAA |
| ZEB1 | qRT-PCR | F: CATCTTGAGCTGAATTTGGGTAACA |
|  |  | R: CCTGAAATGACCTGAAGCATGAA |
| GAPDH | qRT-PCR | F: GCACCGTCAAGGCTGAGAAC |
|  |  | R: TGGTGAAGACGCCAGTGGA |
| Pri-miR-141 | qRT-PCR | F: TGGATCCAGAACCCACAGTC |
|  |  | R: TCTCCTTCCCATTGTTCCCC |
| Pre-miR-141 | qRT-PCR | F: CTGGGTCCATCTTCCAGTACAGTG |
|  |  | R: AGCCATCTTTACCAGACAGTGTTA |
| Pri-miR-200c | qRT-PCR | F: GTGTCAGCAACATCCATCGC |
|  |  | R: GCTCTCAGCTCAAGACGAGG |
| Pre-miR-200c | qRT-PCR | F: AGGGATCTGCAGCTTTTCCG |
|  |  | R: ACCTGAGGCGATGGATGTTG |
| hsa-miR-200a-3p | sqRT-PCR | CGGCTA ACACTGTCTGGTAACGATG |
| hsa-miR-200b-3p | sqRT-PCR | GCGGCGTAATACTGCCTGGTAATG |
| hsa-miR-200c-3p | sqRT-PCR | GCGTAATACTGCCGGGTAATGATGG |
| hsa-miR-429 | sqRT-PCR | GGCGGTAATACTGTCTGGTAAAACCG |
| hsa-miR-141-3p | sqRT-PCR | GCGGCGTAACACTGTCTGGTAAAG |
| hsa-miR-150-5p | sqRT-PCR | GCTCTCCCAACCCTTGTACCAGT |
| hsa-miR-130b-3p | sqRT-PCR | GCCAGTGCAATGATGAAAGGGC |
| hsa-miR-101-3p | sqRT-PCR | GCGCGGCTACAGTACTGTGATAACT |
| hsa-miR-23b-3p | sqRT-PCR | GCCATCACATTGCCAGGGATTACC |
| hsa-miR-205-5p | sqRT-PCR | GCTCCTTCATTCCACCGGAGTC |
| hsa-miR-494-3p | sqRT-PCR | GGCCTGAAACATACACGGGAAACC |
| hsa-miR-539-5p | sqRT-PCR | GCGCGGGAGAAATTATCCTTGGTG |
| hsa-miR-655-3p | sqRT-PCR | GCCGCGGCATAATACATGGTTAACC |
| hsa-miR-1236-3p | sqRT-PCR | GCCTCTTCCCCTTGTCTCTCCA |
| hsa-miR-183-5p | sqRT-PCR | GGCGTATGGCACTGGTAGAATTCAC |
| hsa-miR-96-5p | sqRT-PCR | CGCTTTGGCACTAGCACATTTTTGC |
| hsa-miR-142-5p | sqRT-PCR | GCCGCGCATAAAGTAGAAAGCACT |
| has-miR-591 | sqRT-PCR | GCGCAGACCATGGGTTCTCAT T |
| ELF3 | CHIP-RT-PCR | F: AACCTGGGCCTCGTGATC |
|  |  | R: AACACTGCTGGGTAAGACGA |
| ELF3-down stream | CHIP-qPCR | F: AGGGATCTGCAGCTTTTCCG |
|  |  | R: ACCTGAGGCGATGGATGTTG |
| ELF3-up stream | CHIP-qPCR | F: TAAAGGCCACCAGGGGAGAG |
|  |  | R: GCGACACACACCGATTTACC |
| ZEB1 | CHIP-RT-PCR | CCCAAAGACGTTTCCTTATTCGA |
|  |  | CTCTGAACCTGATAGATTGCTGA |

| Supplementary Table 2. List of the Antibody used in this study | |
| --- | --- |
| Antibody | Source |
| ELF3 | Abcam (ab194943) |
| ELF3-chip | Santa Cruz (sc-376055 x) |
| GAPDH | Byotime (AF0006) |
| β-actin | Byotime (AF0003) |
| E-cadherin | Cell Signaling Technology (14472) |
| N-cadherin | Cell Signaling Technology (13116) |
| Fibronectin | Santa Cruz(sc-8422) |
| ZEB1 | Cell Signaling Technology (3396) |
| ZEB2 | Santa Cruz(sc-271984) |
| Snail | Cell Signaling Technology (C15d3) |
| Slug | Cell Signaling Technology (C19G7) |
| TWIST | Santa Cruz(sc-81417) |

| Supplementary Table 3. The sequence of siRNA, miRNA inhibitor and cDNA clone used in this study | |  |
| --- | --- | --- |
| Name | Sequence |  |
| ELF3-siRNA-1 | CCATGAGGTACTACTACAA |  |
| ELF3-siRNA-2 | GCTACCAAGTGGAGAAGAA |  |
| ELF3-siRNA-3 | CTGTGAGATTAGCAACATT |  |
| ZEB1-siRNA-1 | GGCAAGTGTTGGAGAATAA |  |
| ZEB1-siRNA-2 | GGACAGCACAGTAAATCTA |  |
| ZEB1-siRNA-3 | CGGACGAGAGAGAGAGTTT |  |
| miR-141-3p inhibitor | CCATCTTTACCAGACAGTGTTA |  |
| miR-494-3p inhibitor | GAGGTTTCCCGTGTATGTTTCA |  |
| sequence of ELF3 | ATGGCTGCAACCTGTGAGATTAGCAACATTTTTAGCAACTACTTCAGTGCGATGTACAGCTCGGAGGACTCCACCCTGGCCTCTGTTCCCCCTGCTGCCACCTTTGGGGCCGATGACTTGGTACTGACCCTGAGCAACCCCCAGATGTCATTGGAGGGTACAGAGAAGGCCAGCTGGTTGGGGGAACAGCCCCAGTTCTGGTCGAAGACGCAGGTTCTGGACTGGATCAGCTACCAAGTGGAGAAGAACAAGTACGACGCAAGCGCCATTGACTTCTCACGATGTGACATGGATGGCGCCACCCTCTGCAATTGTGCCCTTGAGGAGCTGCGTCTGGTCTTTGGGCCTCTGGGGGACCAACTCCATGCCCAGCTGCGAGACCTCACTTCCAGCTCTTCTGATGAGCTCAGTTGGATCATTGAGCTGCTGGAGAAGGATGGCATGGCCTTCCAGGAGGCCCTAGACCCAGGGCCCTTTGACCAGGGCAGCCCCTTTGCCCAGGAGCTGCTGGACGACGGTCAGCAAGCCAGCCCCTACCACCCCGGCAGCTGTGGCGCAGGAGCCCCCTCCCCTGGCAGCTCTGACGTCTCCACCGCAGGGACTGGTGCTTCTCGGAGCTCCCACTCCTCAGACTCCGGTGGAAGTGACGTGGACCTGGATCCCACTGATGGCAAGCTCTTCCCCAGCGATGGTTTTCGTGACTGCAAGAAGGGGGATCCCAAGCACGGGAAGCGGAAACGAGGCCGGCCCCGAAAGCTGAGCAAAGAGTACTGGGACTGTCTCGAGGGCAAGAAGAGCAAGCACGCGCCCAGAGGCACCCACCTGTGGGAGTTCATCCGGGACATCCTCATCCACCCGGAGCTCAACGAGGGCCTCATGAAGTGGGAGAATCGGCATGAAGGCGTCTTCAAGTTCCTGCGCTCCGAGGCTGTGGCCCAACTATGGGGCCAAAAGAAAAAGAACAGCAACATGACCTACGAGAAGCTGAGCCGGGCCATGAGGTACTACTACAAACGGGAGATCCTGGAACGGGTGGATGGCCGGCGACTCGTCTACAAGTTTGGCAAAAACTCAAGCGGCTGGAAGGAGGAAGAGGTTCTCCAGAGTCGGAAC |  |
| Supplementary Table 4. The sequence of miR-141 promoter and mutant promoter used in this study | | |
| Name | Sequence | |
| sequence of miR-141 promoter | CTTCTTTATTTGAAACACTGGTGTCCTGGGGAGTAAAGCCGGTGGGAGTCATCCCTCAGGAAGTGCTGGCGCCCACTCCTGGAAAGGCTGAGACAGCACAGGTCCCAAAGCCCAGAGGCTGGGCGTGCATTACTCAGCAAATCCTTACAGAGCCCCCGGCGTCACAGGCATTCACAGTCCCCCGACCTCCTGGAACTTAGGAGGCTGGTCAGGGAGACAGATTCACAAACCGATCACAAGCATCAAATAATTGCAGGCGGGTATTAAGAAGGAAGCAAACAAAGCCTGGGAGAGAGAACAGCGAGGGATTGAGCAACCCAATAGCTCCCTGGGGCTGTGGCCCTCCAGGGCACGGGTGGGGCAATCACCTGTCGGGCCCAGGTCCCCTCTCCCAGCAACGCCCTTTCTATACAAGCCGCAGCTGCACAAAGGCAAGTCCCACCTCCTCTAACCGCCCTTGGGCTACCTGCCCTTGGGGTGGGACTTGGACTCCACTGAGGGCTGTGCTGTGAGGTGGGTCCGGGAGCAGCTCGGTCCGGAGAGTGGAGCGCGATCGTTCCCTCTGCAGGCCTCAATCGAGGGGCAAGGCTGATGCTCCGGGCCAGCTGGGGTCTCTGGGTAGCGGTGGGTAACTTCACACTAGCGACACCTTGCTGGGACCCGCCTGCCCTTCACAGGCCTGGCGGGGCCTTCTCCCTCCCCTTTCCCTCAGGGGATCCCAGCACAGGCTGGGCACTGCGGGGCGGCACAGCCCAACTCCTGCCCAGCTGACCCCTCGCTGACCTCAGGGATCTCTCTGGCCTGCAGCTCCGCTGTGGGCAGGGTCTGAGGCCACAGAGGAATGGGCTAGTCCTGGGGGCAGCATCTGCTGTGGGGAGGGGACCCAAGGACAGCCCCCGCTTTTTGTACCTCTGGAGACAGGGGTGAGACTAGGCAGGTTGGAGAAAAGAGGCCCCTGGGAGAGGGTGGGAGGCCTAGAGGAGTGGCCAAGCCTTAGAGGAGGTGCCCGTGGCTGGCGCTGGGAGGGAAGGGGTTAAGGCAGTGGGGGGGCAGCCTATGGCAGGAGGACACACCTGTGCGCAGGGTGGCAGGCGGGGCCCAGGTAAGGAGCCTGCGCTGGCTGCCCGGCAGGCGGAGAAGGAAGGAGGAAGAGCGGAGGCCAGGGCGGGCTCTAGGCCGTGGAATCTGGGGCCTTAAAGCCCCTTCGTCTCCCCAGCACCCACTCTCTGGGGGCAGGTGGGCCCGGTGACAGGTAAAGGCCACCAGGGGAGAGGTCCTGGGCTGAGCTTGGGACTGCAGAGGGGGGATGAGGGTGGGTAAATCGGTGTGTGTCGCGGGTCGGGAAAGGCTGCCGGGGGTAGGGGAAGGTGGCTCAGAGGCGGCGGGCCGACGGTCGAGGGGCTTCGGAGGGCCTGCTTGGACTGCAACCTGGGCCTCGTGATCAGCGACCCAGGGTGTGGCTGGTGGCGGGCAGCAGGGCTCACCAGGAAGTGTCCCCAGGGACTCGGGTGGTGGGGGGATGGGAGCCAGGGATCTGCAGCTTTTCCGCAGGGATCCTGGGCCTGAAGCTGCCTGACCCAAGGTGGGCGGGCTGGGCGGGGGCCCTCGTCTTACCCAGCAGTGTTTGGGTGCGGTTGGGAGTCTCTAATACTGCCGGGTAATGATGGAGGCCCCTGTCCCTGTGTCAGCAACATCCATCGCCTCAGGTCCCCAGCCCTTAGCTGGCTGCAGCCCCCTCCCCACTTCCCACGCACCCCGGAAGCCCCTCGTCTTGAGCTGAGAGCGTTGCACAAGGGGTGGTTCTTGTTGGCTGGCTGCCACTAAGGGACACAATGGGCCCCAGCCCCTCCTCCCACCCAGTGCGATTTGTCACCTGGTGGATCCAGAACCCACAGTCGACCTTGAGCTTGGGGTTGGCTCGCCCCCTCTCAAGAGACCTCACCTGGCCTGTGGCCAGGGTCCCCTGTAGCAACTGGTGAGCGCGCACCGTAGTTCTCTGT | |
| sequence of miR-141 mutant promoter | CTTCTTTATTTGAAACACTGGTGTCCTGGGGAGTAAAGCCGGTGGGAGTCATCCCTCAGGAAGTGCTGGCGCCCACTCCTGGAAAGGCTGAGACAGCACAGGTCCCAAAGCCCAGAGGCTGGGCGTGCATTACTCAGCAAATCCTTACAGAGCCCCCGGCGTCACAGGCATTCACAGTCCCCCGACCTCCTGGAACTTAGGAGGCTGGTCAGGGAGACAGATTCACAAACCGATCACAAGCATCAAATAATTGCAGGCGGGTATTAAGAAGGAAGCAAACAAAGCCTGGGAGAGAGAACAGCGAGGGATTGAGCAACCCAATAGCTCCCTGGGGCTGTGGCCCTCCAGGGCACGGGTGGGGCAATCACCTGTCGGGCCCAGGTCCCCTCTCCCAGCAACGCCCTTTCTATACAAGCCGCAGCTGCACAAAGGCAAGTCCCACCTCCTCTAACCGCCCTTGGGCTACCTGCCCTTGGGGTGGGACTTGGACTCCACTGAGGGCTGTGCTGTGAGGTGGGTCCGGGAGCAGCTCGGTCCGGAGAGTGGAGCGCGATCGTTCCCTCTGCAGGCCTCAATCGAGGGGCAAGGCTGATGCTCCGGGCCAGCTGGGGTCTCTGGGTAGCGGTGGGTAACTTCACACTAGCGACACCTTGCTGGGACCCGCCTGCCCTTCACAGGCCTGGCGGGGCCTTCTCCCTCCCCTTTCCCTCAGGGGATCCCAGCACAGGCTGGGCACTGCGGGGCGGCACAGCCCAACTCCTGCCCAGCTGACCCCTCGCTGACCTCAGGGATCTCTCTGGCCTGCAGCTCCGCTGTGGGCAGGGTCTGAGGCCACAGAGGAATGGGCTAGTCCTGGGGGCAGCATCTGCTGTGGGGAGGGGACCCAAGGACAGCCCCCGCTTTTTGTACCTCTGGAGACAGGGGTGAGACTAGGCAGGTTGGAGAAAAGAGGCCCCTGGGAGAGGGTGGGAGGCCTAGAGGAGTGGCCAAGCCTTAGAGGAGGTGCCCGTGGCTGGCGCTGGGAGGGAAGGGGTTAAGGCAGTGGGGGGGCAGCCTATGGCAGGAGGACACACCTGTGCGCAGGGTGGCAGGCGGGGCCCAGGTAAGGAGCCTGCGCTGGCTGCCCGGCAGGCGGAGAAGGAAGGAGGAAGAGCGGAGGCCAGGGCGGGCTCTAGGCCGTGGAATCTGGGGCCTTAAAGCCCCTTCGTCTCCCCAGCACCCACTCTCTGGGGGCAGGTGGGCCCGGTGACAGGTAAAGGCCACCAGGGGAGAGGTCCTGGGCTGAGCTTGGGACTGCAGAGGGGGGATGAGGGTGGGTAAATCGGTGTGTGTCGCGGGTCGGGAAAGGCTGCCGGGGGTAGGGGAAGGTGGCTCAGAGGCGGCGGGCCGACGGTCGAGGGGCTTCGGAGGGCCTGCTTGGACTGCAACCTGGGCCTCGTGATCAGCGACCCAGGGTGTGGCTGGTGGCGGGCAGCAGGGCTCACCACCTTGTGTCCCCAGGGACTCGGGTGGTGGGGGGATGGGAGCCAGGGATCTGCAGCTTTTCCGCAGGGATCCTGGGCCTGAAGCTGCCTGACCCAAGGTGGGCGGGCTGGGCGGGGGCCCTCGTCTTACCCAGCAGTGTTTGGGTGCGGTTGGGAGTCTCTAATACTGCCGGGTAATGATGGAGGCCCCTGTCCCTGTGTCAGCAACATCCATCGCCTCAGGTCCCCAGCCCTTAGCTGGCTGCAGCCCCCTCCCCACTTCCCACGCACCCCGGAAGCCCCTCGTCTTGAGCTGAGAGCGTTGCACAAGGGGTGGTTCTTGTTGGCTGGCTGCCACTAAGGGACACAATGGGCCCCAGCCCCTCCTCCCACCCAGTGCGATTTGTCACCTGGTGGATCCAGAACCCACAGTCGACCTTGAGCTTGGGGTTGGCTCGCCCCCTCTCAAGAGACCTCACCTGGCCTGTGGCCAGGGTCCCCTGTAGCAACTGGTGAGCGCGCACCGTAGTTCTCTGT | |

**Legends to Supplementary Figures**

Supplementary Figure 1:

(A) subcutaneous xenograft tumor assay showed that ELF3 knockdown inhibits tumor growth, tumor volumes of tumors were shown in the right panel. (B) protein expression of ZEB2, SLUG, SNAIL, and ELF3 in MHCC-LM3-shELF3 cells, Huh7-ELF3 cells and their control cells were analyzed by western blot. (C) ZEB1 expression positively correlated with ELF3 expression according to TCGA database from GEPIA. (D) predicted ELF3 binding site in ZEB1 promoter and CHIP assay. (E) Relative expression of miRNAs in Huh7-ELF3 cells and Huh7-control cells were analyzed by sqRT-PCR. (F) protein expression of ZEB1 and E-cadherin in Huh7-ELF3 cells and Huh7-control cells with miR-141-3p inhibitor and miR-141-3p inhibitor control were analyzed by western blot. P<0.05 was considered statistically significant. Each error bar represents the mean± SD of three replicate samples. P<0.05 was considered statistically significant. * p<0.05, **p<0.01 and *** p<0.001 based on the Student t test.

Supplementary Figure 2:

(A) migration and invasion assay showed that miR-141-3p inhibitor increase MHCC-LM3 cells migration and invasion (magnification ×100, scale bar:200μm). (B) relative pri-miRNA-141 expression in MHCC-LM3 cell, Huh7-ELF3 cells, and their control cells were analyzed by qPCR. (C) relative pre-miRNA-141 expression in MHCC-LM3 cell, Huh7-ELF3 cells, and their control cells were analyzed by qPCR. (D) relative pri-miRNA-200c expression in MHCC-LM3 cell, Huh7-ELF3 cells, and their control cells were analyzed by qPCR. (E) relative pre-miRNA-200c expression in MHCC-LM3 cell, Huh7-ELF3 cells, and their control cells were analyzed by qPCR. (F) Chip qPCR assay in MHCC-LM3 cells with or without knocking down ELF3 for the genomic regions upstream and downstream of ELF3 binding sites as negative controls. P<0.05 was considered statistically significant. Each error bar represents the mean± SD of three replicate samples. P<0.05 was considered statistically significant. * p<0.05, **p<0.01 and *** p<0.001 based on the Student t test.
