# Supplementary figures and images for: ELF3 promotes epithelial–mesenchymal transition by protecting ZEB1 from miR-141-3p-mediated silencing in hepatocellular carcinoma
Source: Cell Death Dis. 2018 Mar 9;9(3):387. doi: 10.1038/s41419-018-0399-y (PMC5845010; doi:10.1038/s41419-018-0399-y)

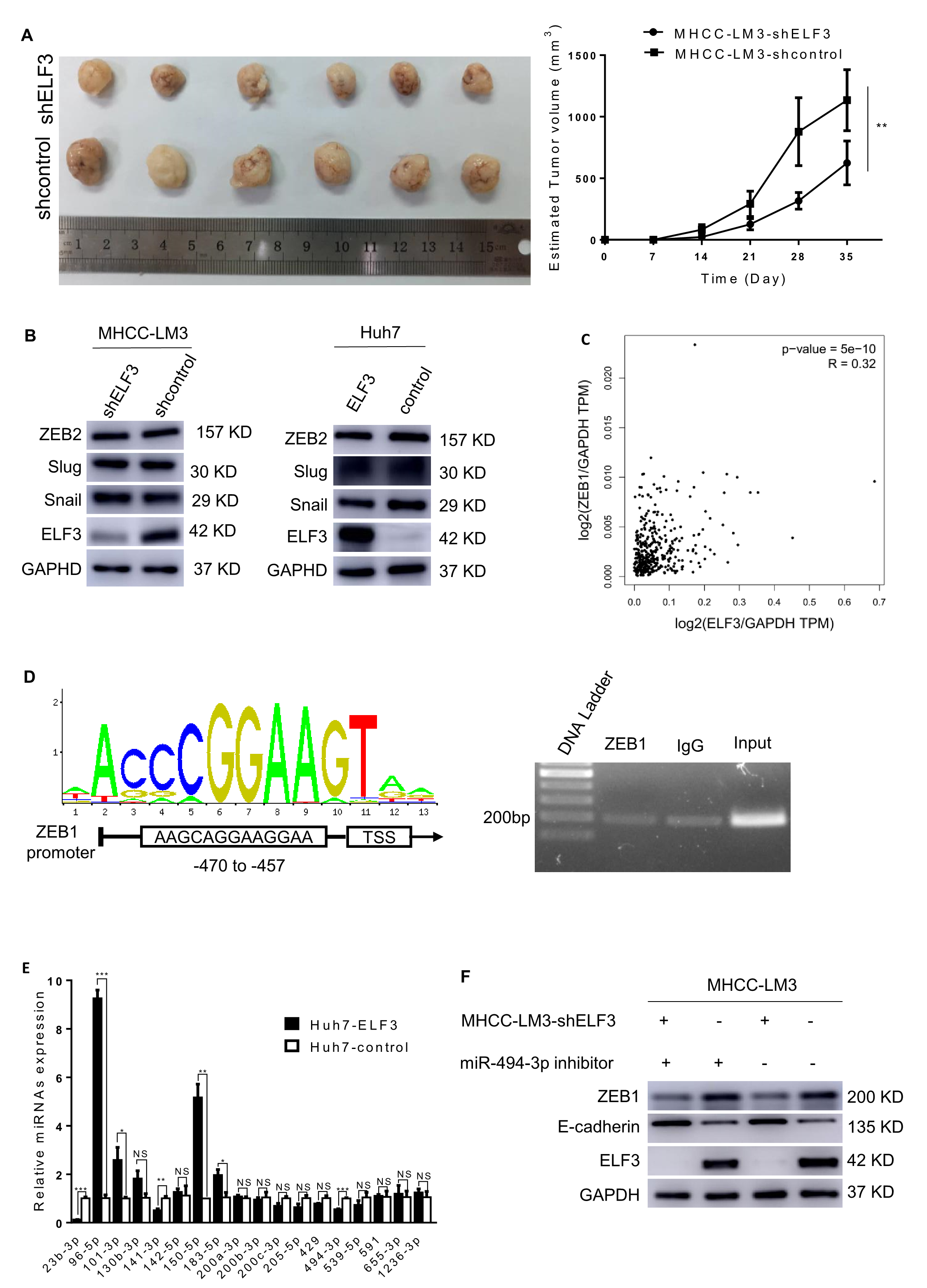

Supplement: Supplementary file 2 — Supplementary Figure 1 [file 41419_2018_399_MOESM2_ESM.tif]

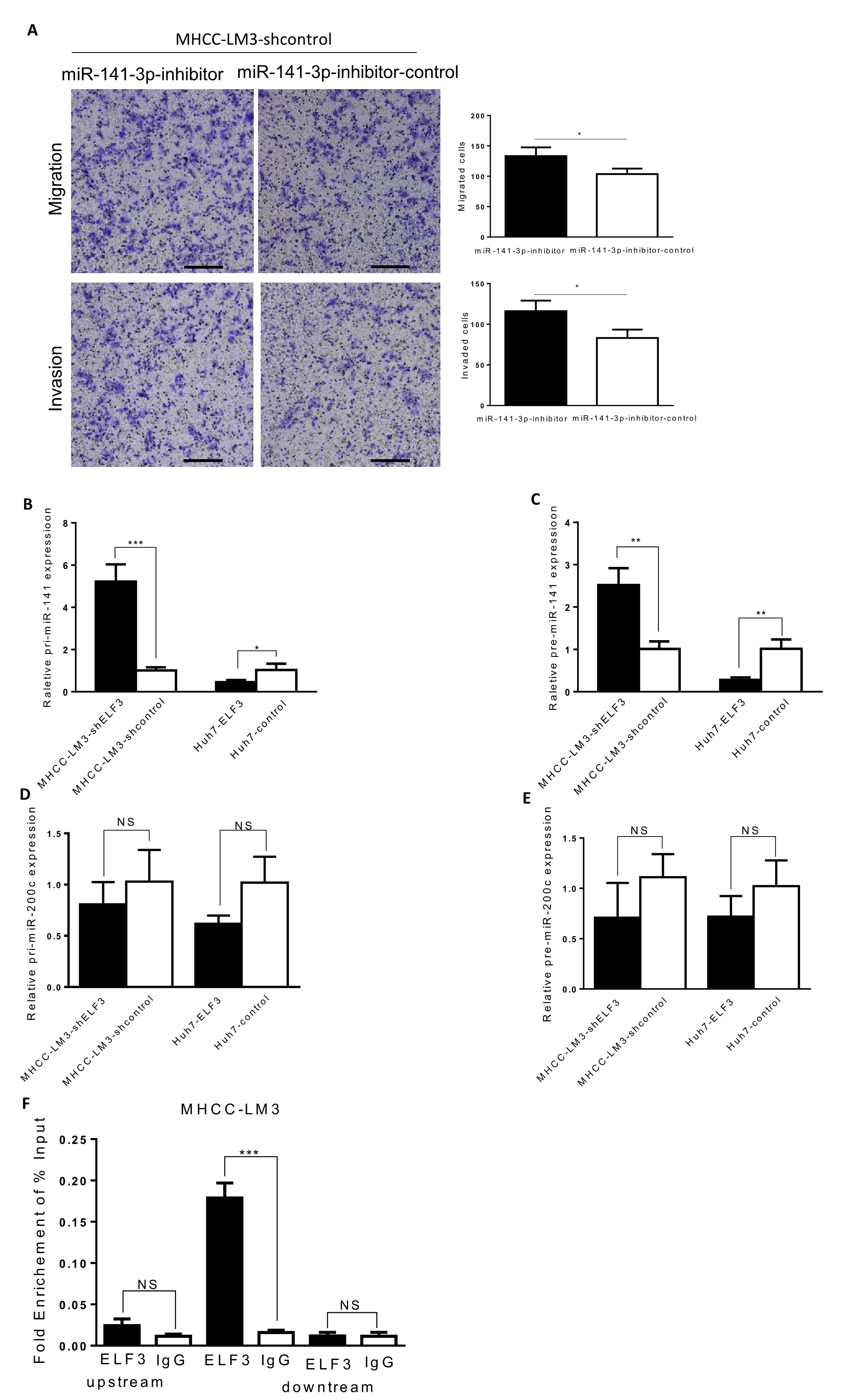

Supplement: Supplementary file 3 — Supplementary Figure 2 [file 41419_2018_399_MOESM3_ESM.tif]
